# Correction: Hydrophobic mesoporous silicon dioxide for improving foam stability

**DOI:** 10.1039/d0ra90065f

**Published:** 2020-06-09

**Authors:** Lihui Meng, Qingwang Liu, Jigang Wang, Zhenzhong Fan, Xiaoming Wei

**Affiliations:** Key Laboratory of Improving Oil Recovery by Ministry of Education, Northeast Petroleum University Daqing Heilongjiang 163111 China liuqingwang@163.com; National Energy Research and Development Center of Heavy Oil Panjin Liaoning 124000 China

## Abstract

Correction for ‘Hydrophobic mesoporous silicon dioxide for improving foam stability’ by Lihui Meng *et al.*, *RSC Adv.*, 2020, **10**, 18565–18571, DOI: 10.1039/D0RA02161J.

The authors regret that an incorrect grant number was shown in the acknowledgements section of the published article. The corrected section should read:

The authors acknowledge the support from National Science and Technology Major Project-Research on Hot Air Foam Composite Flooding Technology after Steam Flooding, 2016ZX05055.

The Royal Society of Chemistry apologises for these errors and any consequent inconvenience to authors and readers.

## Supplementary Material

